# Primary malignant melanoma of the pancreas diagnosed by endoscopic ultrasonography-guided fine-needle biopsy

**DOI:** 10.1055/a-2092-0107

**Published:** 2023-06-12

**Authors:** Shenglin Xu, Yaping Ye, Wen Guo, Side Liu, Yue Li

**Affiliations:** 1 Department of Gastroenterology, Nanfang Hospital, Southern Medical University, Guangzhou, P. R. China; 2 Department of Pathology, Nanfang Hospital, Southern Medical University, Guangzhou, P. R. China

Endoscopic ultrasonography-guided fine-needle biopsy (EUS-FNB) plays an important role in obtaining pathology specimens from pancreatic lesions. FNB needles have been designed to acquire larger “core” specimens that preserve tissue architecture and permit histologic evaluation and immunohistologic staining 1 2 . In this study, we report an extremely rare case of primary pancreatic melanoma diagnosed by EUS-FNB using a 22-gauge Franseen needle.

A 59-year-old woman presented with a 2-day history of abdominal pain. Her laboratory tests including liver function, pancreatic amylase, and tumor markers were all within normal limits. Computed tomography revealed a low-density round lesion measuring 4.5 × 5.1 cm in the head of the pancreas with invasion into the portal vein ( Fig. 1 ). Positron emission tomography/computed tomography showed a cystic tumor at the head of the pancreas with a moderate level of glucose metabolism ( Fig. 2 ). To determine the diagnosis, we carried out EUS-FNB of the pancreatic mass ( Fig. 3 a , Video 1 ) and obtained a large amount of black tissue ( Fig. 3 b ). Hematoxylin and eosin staining showed atypical tumor cells accompanied by melanin deposition ( Fig. 4 a ); immunohistochemically, the tumor cells were positive for melanocytic marker Human Melanoma Black 45 (HMB-45) ( Fig. 4 b ) and Melan-A. The pathological findings revealed malignant melanoma of the pancreas, and we carefully re-checked that there were no pigmented spots on the skin or oral mucosa. Eventually, the patient was diagnosed with primary melanoma of the pancreas. She has received regular toripalimab immunotherapy combined with anlotinib, and after two cycles of therapy her abdominal pain was significantly relieved.

**Fig. 1:**
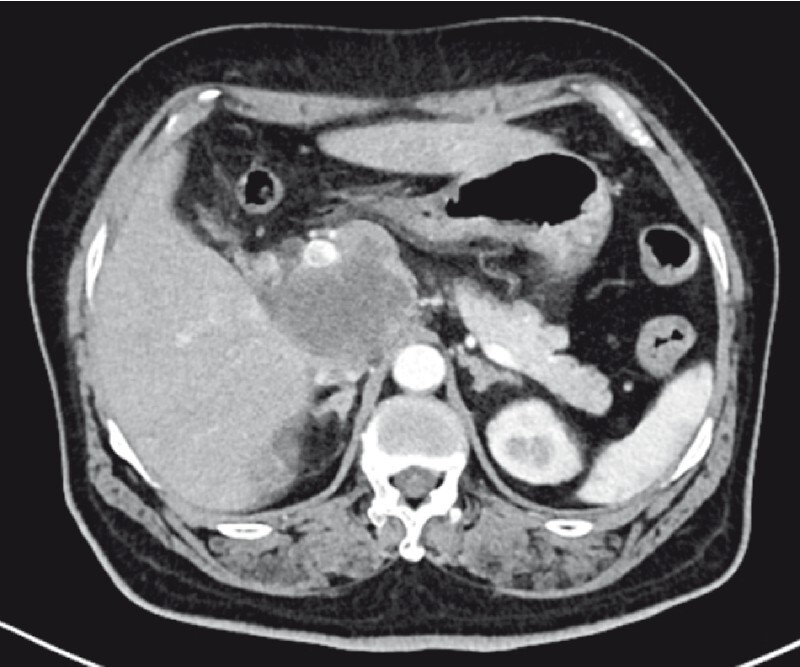
Computed tomography revealed a mass in the head of the pancreas.

**Fig. 2:**
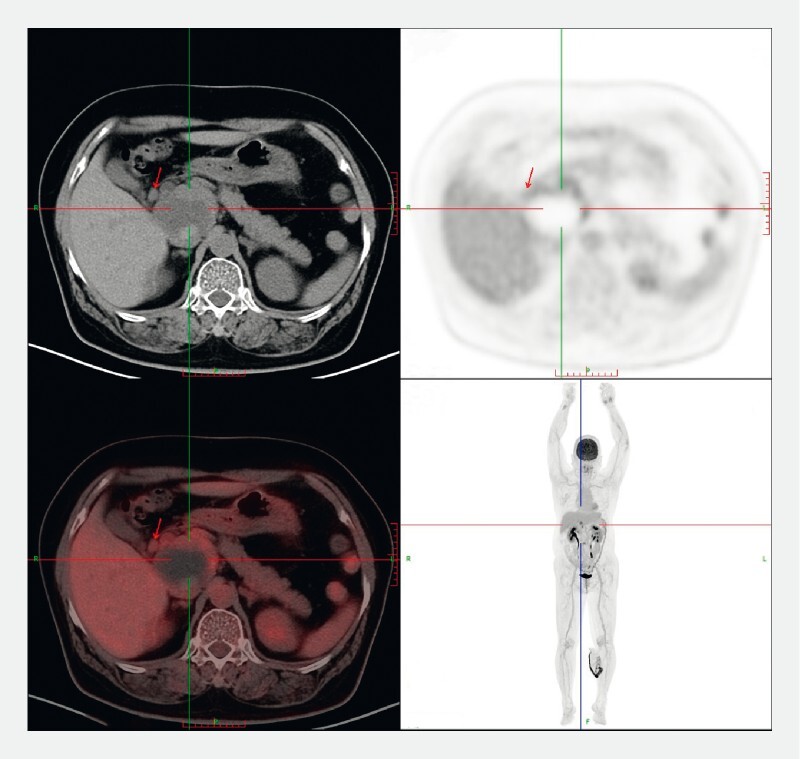
Positron emission tomography/computed tomography of the mass.

**Fig. 3 a:**
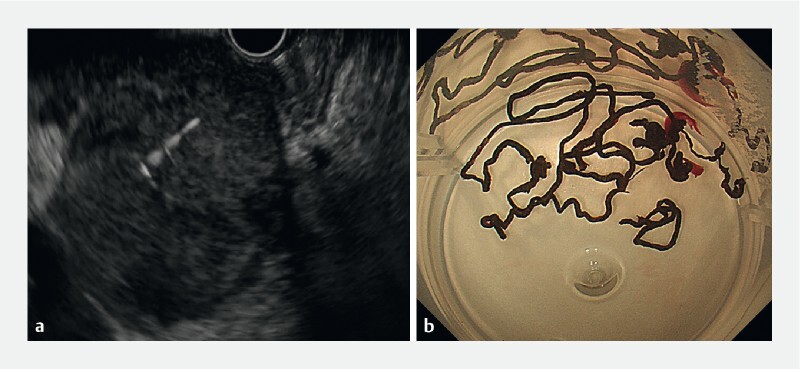
Endoscopic ultrasonography showed a low-density round lesion measuring 4.5 × 5.1 cm with regular borders in the head of the pancreas. **b** Black tissue obtained by endoscopic ultrasonography-guided fine-needle biopsy.

**Video 1** Endoscopic ultrasonography-guided fine-needle biopsy of primary malignant melanoma of the pancreas.


**Fig. 4:**
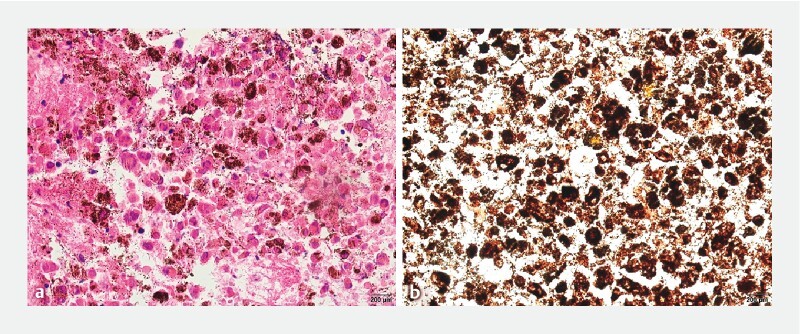
Histopathology of the pancreatic tissue samples. **a** Hematoxylin and eosin staining showed atypical tumor cells accompanied by melanin deposition (magnification, × 400). **b** Immunohistochemical staining was positive for Human Melanoma Black 45 (magnification, × 400).

To our knowledge, this is the first case of primary malignant melanoma of the pancreas diagnosed using the EUS-FNB technique. As our case has demonstrated, EUS-FNB was crucial for the tissue acquisition and pathological diagnosis.

Endoscopy_UCTN_Code_CCL_1AZ_2AB

## Notes

Endoscopy E-Videos

https://eref.thieme.de/e-videos
*E-Videos* is an open access online section of the journal *Endoscopy* , reporting on interesting cases and new techniques in gastroenterological endoscopy. All papers include a high-quality video and are published with a Creative Commons CC-BY license. Endoscopy E-Videos qualify for HINARI discounts and waivers and eligibility is automatically checked during the submission process. We grant 100% waivers to articles whose corresponding authors are based in Group A countries and 50% waivers to those who are based in Group B countries as classified by Research4Life (see: https://www.research4life.org/access/eligibility/ ). This section has its own submission website at https://mc.manuscriptcentral.com/e-videos

## References

[1] Polkowski M Jenssen C Kaye P Technical aspects of endoscopic ultrasound (EUS)-guided sampling in gastroenterology: European Society of Gastrointestinal Endoscopy (ESGE) Technical Guideline March 2017 Endoscopy 2017 49 989 1006 28898917 10.1055/s-0043-119219

[2] Bang J Y Hebert-Magee S Hasan M K Endoscopic ultrasonography-guided biopsy using a Franseen needle design: initial assessment Dig Endosc 2017 29 338 346 27878861 10.1111/den.12769

